# Comparison of alfentanil and sufentanil on extubation-related cough following suspension laryngoscopy surgery: a randomized controlled trial

**DOI:** 10.3389/fmed.2026.1863301

**Published:** 2026-07-17

**Authors:** Qiang Wei, Jiaman Li, Chunyang Shao, Yan Tang, Yifeng Yang

**Affiliations:** Anesthesiology Operating Center, West China Hospital of Sichuan University–Ziyang Hospital (Ziyang Central Hospital), Ziyang, Sichuan, China

**Keywords:** alfentanil, cough, extubation cough, sufentanil, suspension laryngoscopy surgery

## Abstract

**Introduction:**

This study aimed to compare the effects of alfentanil and sufentanil on cough response during tracheal extubation in patients undergoing suspension laryngoscopy surgery.

**Methods:**

In this prospective, randomized controlled trial, 80 patients (ASA I–II) scheduled for elective suspension laryngoscopy were enrolled. Patients were randomly allocated to receive either alfentanil (Group A, *n* = 42; The induction dose for general anesthesia is 21 μg/kg, and maintenance 0.7–1.4 μg·kg^−1^·min^−1^) or sufentanil (Group S, *n* = 38; The induction dose for general anesthesia is 0.3 μg/kg, and maintenance 0.01–0.02 μg·kg^−1^·min^−1^), both combined with propofol 2–4 μg·mL^−1^ target-controlled infusion. The primary outcome was the incidence of cough, including moderate-to-severe cough within 5 min after tracheal extubation. Secondary outcomes included cough during induction, hemodynamic variables, extubation time, recovery scores, postoperative pain, and adverse events.

**Results:**

The incidence of cough within 5 min after tracheal extubation was significantly higher in Group A than in Group S (69.0% [29/42] vs. 44.7% [17/38]; OR 2.76, 95%CI 1.08–7.00; *p* = 0.03), whereas the incidence of moderate-to-severe cough did not differ between groups (*p* > 0.05). During induction, cough occurred less frequently in Group A (11.9% [5/42] vs. 31.6% [12/38]; OR 0.29, 95%CI 0.10–0.89; *p* = 0.03). Heart rate and mean arterial pressure were lower in Group A at specific intraoperative time points (*p* < 0.05). Extubation time, Aldrete scores, and postoperative pain scores were comparable between groups (*p* > 0.05). No opioid-related adverse events were observed.

**Conclusion:**

In suspension laryngoscopy surgery, alfentanil is associated with a lower incidence of cough during induction but a higher incidence of cough during extubation compared with sufentanil. There was no statistically significant difference in the incidence of moderate-to-severe cough between alfentanil and sufentanil.

## Introduction

Suspension laryngoscopy is a minimally invasive procedure commonly performed for laryngopharyngeal diseases. Although the surgical duration is typically short, the procedure involves intense intraoperative stimulation. The incidence of cough during the emergence and extubation phase is reported to be as high as 50–70% (1, 2). Extubation-related cough can precipitate severe complications, including acute hypertension, tachycardia, elevated intracranial pressure, and bleeding at the surgical site (3). Sufentanil is one of the most widely used opioid analgesics in clinical general anesthesia practice, yet its application is complicated by a considerably high incidence of cough during the induction of anesthesia (4). Alfentanil is a short-acting opioid with lower lipid solubility than fentanyl and sufentanil. It is characterized by a rapid onset of action (plasma-effect site equilibration half-life of 0.6–1.0 min) and rapid clearance (elimination half-life of 90 min). As a short-acting opioid, alfentanil can provide adequate intraoperative analgesia while reducing the risk of residual respiratory depression during anesthetic emergence (5). Previous studies have shown that alfentanil effectively reduces the incidence of cough during the induction of general anesthesia while preserving hemodynamic stability (6). However, data on the effect of alfentanil on the cough response during tracheal extubation in patients undergoing suspension laryngoscopy remain limited. For perioperative cough in suspension laryngoscopy surgery, preventing cough at extubation is more important than preventing cough during induction. During induction, neuromuscular blocking agents can promptly resolve the cough issue associated with general anesthesia induction, but they are no longer applicable during the extubation phase. Therefore, this study aimed to compare alfentanil with sufentanil to investigate the effect of alfentanil on the cough response during tracheal extubation in this patient population.

## Subjects and methods

### Study population

**Figure 1:**
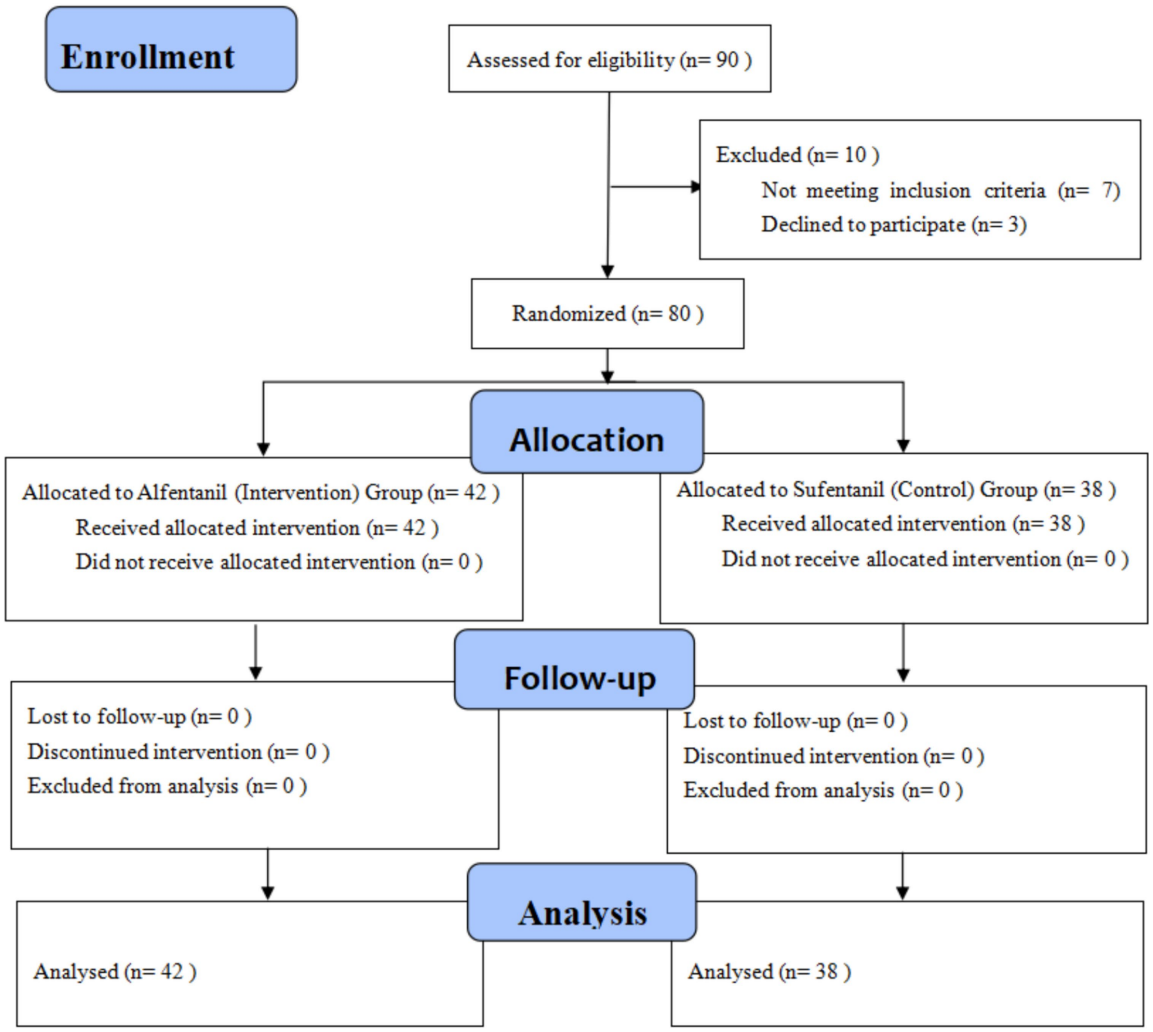
CONSORT flow diagram showing patient enrollment, allocation, follow-up, and analysis.

This was a prospective, randomized controlled trial conducted at Ziyang Central Hospital between July 2025 and December 2025. This trial was prospectively registered with the Chinese Clinical Trial Registry (ChiCTR2500104073^1^) on June 10, 2025, prior to the enrollment of the first patient. The registered outcome (incidence and severity of cough after tracheal extubation) is identical to the primary outcome reported in this manuscript. No substantive modifications to the primary endpoint were made after trial registration. A total of 90 patients scheduled for elective suspension laryngoscopy under general anesthesia were assessed for eligibility in this study. Among them, 10 patients were excluded (seven did not meet the inclusion criteria, and three declined to participate). Finally, 80 eligible patients were randomized and formally enrolled in the study. This study protocol was approved by the Ethics Committee of Ziyang Central Hospital (Approval No. 2024-ky-285), and written informed consent was obtained from all subjects prior to surgery. The CONSORT flow diagram is presented in Figure 1.

Inclusion criteria: (1) Age 18–65 years; (2) American Society of Anesthesiologists (ASA) physical status I–II; and (3) Body mass index (BMI) 18–27 kg/m^2^.

Exclusion criteria: (1) Difficult airway or anticipated difficulty with intubation; (2) Severe cardiac, pulmonary, hepatic, or renal insufficiency; (3) Poorly controlled hypertension (systolic blood pressure >160 mmHg or diastolic blood pressure >100 mmHg); (4) Chronic use of opioids or sedative-hypnotics; (5) Known allergy to propofol, opioids, or other study medications; (6) History of psychiatric illness or communication barriers precluding cooperation with the study.

### Sample size calculation

In a pilot study involving 10 patients in each group, the incidence of cough during extubation was 80% (8/10) in the alfentanil group and 50% (5/10) in the sufentanil group. With a two-sided significance level of *α* = 0.05 and a power of 1–*β* = 0.80, the sample size required was calculated to be 36 patients per group using PASS software (version 25.0.5). To account for a potential dropout rate of 10%, 40 patients per group were required, resulting in a total sample size of 80 patients.

### Randomization and blinding

Patients were randomly allocated to either the alfentanil group (Group A, *n* = 42) or the sufentanil group (Group S, *n* = 38) using a random number table. Allocation concealment was maintained using sealed, opaque envelopes managed by a research assistant. All study drugs (alfentanil and sufentanil injections) were prepared and packaged by an anesthesia nurse who was not involved in data collection, according to the randomization codes. Syringes were labeled only with the patient identification number and group code. All outcome assessments were performed by investigators who were blinded to group assignment.

### Anesthetic management

On arrival in the operating room, standard anesthesia monitoring was instituted, including continuous electrocardiogram (ECG), noninvasive blood pressure (NIBP), pulse oximetry (SpO₂), and bispectral index (BIS) monitoring. A peripheral intravenous catheter was placed, and lactated Ringer’s solution was administered via intravenous infusion.

#### Anesthesia induction protocol

Equianalgesic dosing of the study opioids was determined based on validated pharmacodynamic data from prior literature (7): the analgesic potency of sufentanil is approximately 10-fold that of fentanyl, while alfentanil has approximately 1/7 the analgesic potency of fentanyl, resulting in an estimated 70:1 equianalgesic potency ratio of sufentanil to alfentanil.

Alfentanil Intervention Group (Group A): After baseline hemodynamic stabilization, patients were administered 21 μg/kg of alfentanil via slow intravenous bolus over no less than 30 s. This was followed by target-controlled infusion (TCI) of propofol, titrated to an effect-site concentration of 3.5 μg/mL with the Marsh pharmacokinetic model. Once loss of consciousness (LOC) was confirmed, 0.6 mg/kg of rocuronium bromide was given intravenously to facilitate tracheal intubation.

Sufentanil Control Group (Group S): Patients received a slow intravenous bolus of 0.3 μg/kg sufentanil over no less than 30 s. All other induction procedures, including propofol TCI settings and neuromuscular blockade administration, were completely consistent with the Alfentanil Group to eliminate confounding variables. After complete neuromuscular blockade was achieved, tracheal intubation was performed by an experienced anesthesiologist using a video laryngoscope. Correct intratracheal placement of the endotracheal tube was verified by bilateral auscultation of symmetric, equal breath sounds in both lung fields prior to formal fixation of the tube.

Anesthesia Maintenance: After successful tracheal intubation, anesthesia was maintained with protocolized intravenous infusions for both groups, with all dosing adjusted to maintain standardized anesthesia depth and hemodynamic targets:

Alfentanil Intervention Group (Group A): Continuous target-controlled infusion (TCI) of propofol using the Marsh pharmacokinetic model, with the effect-site concentration titrated between 2 and 4 μg/mL, in combination with continuous intravenous alfentanil infusion at 0.7–1.4 μg·kg^−1^·min^−1^ via a calibrated syringe pump.

Sufentanil Control Group (Group S): Propofol TCI with identical pharmacokinetic model and concentration settings to Group A, in combination with continuous intravenous sufentanil infusion at 0.01–0.02 μg·kg^−1^·min^−1^ via a calibrated syringe pump.

#### Intraoperative hemodynamic and anesthesia depth control

Bispectral index (BIS) was continuously monitored throughout the procedure, with values maintained within the target range of 40–60 to avoid intraoperative awareness and excessive anesthetic depth. For deviations in BIS beyond the target range, the propofol TCI effect-site concentration was adjusted in a stepwise manner. Hemodynamic stability was defined as blood pressure fluctuations within 20% of the preoperative baseline; for sustained deviations beyond this range, rescue intravenous boluses of ephedrine (3–9 mg per dose) were administered, with additional vasoactive agents available as needed.

#### Standardized emergence and extubation protocol

Upon completion of the surgical procedure, all anesthetic infusions were immediately discontinued. Oropharyngeal suctioning was performed in a standardized manner to clear all residual blood, mucus, and secretions without excessive airway stimulation. Residual neuromuscular blockade was confirmed via train-of-four (TOF) monitoring, and reversed with intravenous neostigmine 0.02 mg/kg and atropine 0.01 mg/kg. Tracheal extubation was performed only when patients met all pre-specified extubation criteria: (1) full recovery of spontaneous ventilation with a tidal volume ≥6 mL/kg and respiratory rate 10–20 breaths/min; (2) ability to follow simple verbal commands; (3) sustained SpO₂ ≥ 95% on room air. All patients were transferred to the post-anesthesia care unit (PACU) for continuous standardized monitoring after uneventful extubation.

## Outcome measures

### Primary outcome

The incidence and severity of cough within 5 min after tracheal extubation. Cough severity was graded according to the criteria described by Minogue et al. (8): Grade 0 = no cough; Grade 1 = single cough; Grade 2 = cough lasting ≤5 s; Grade 3 = cough lasting >5 s with body movement. Grades 1–3 were defined as the presence of a cough response, and Grades 2–3 were defined as moderate-to-severe cough.

### Secondary outcomes

The incidence and severity of cough during induction of general anesthesia (from opioid administration to loss of consciousness). Hemodynamic parameters: Mean arterial pressure (MAP) and heart rate (HR) recorded at the following time points: upon arrival in the operating room (T0), before intubation (T1), 1 min after intubation (T2), 3 min after intubation (T3), 1 min after suspension laryngoscope placement (T4), 3 min after suspension laryngoscope placement (T5), and at extubation (T6). Postoperative recovery: Extubation time (interval from discontinuation of anesthetics to extubation) and modified Aldrete scores on admission to and discharge from the PACU. Throat pain: Numerical Rating Scale (NRS) scores (0 = no pain, 10 = worst pain imaginable) assessed on PACU discharge and at 1 h postoperatively. Adverse events: Postoperative wound bleeding, postoperative nausea and vomiting, respiratory depression (pulse oxygen saturation <90% for >30 s), chest wall rigidity, pruritus, and urinary retention.

## Statistical analysis

Statistical analyses were performed using SPSS software (version 26.0). Normally distributed continuous data are presented as mean ± standard deviation (x̄ ± s) and were compared between groups using the independent samples *t*-test. Repeated measures data were analyzed using repeated measures analysis of variance (ANOVA); Given that the two groups in this study had similar sample sizes and the total sample size was greater than 30, when the covariance matrices were not homogeneous, we reported the multivariate test results (Pillai’s trace), which is robust to heterogeneity of covariance matrices. Additionally, a linear mixed-effects model with an unstructured covariance structure was fitted as a sensitivity analysis to verify the robustness of the main findings. If an interaction effect was detected, simple effects analysis was performed. Non-normally distributed continuous data are presented as median (Q1, Q3) and were compared using the Mann–Whitney U test. Categorical data are presented as numbers (%) and were compared using the chi-square (*χ*^2^) test or Fisher’s exact test, as appropriate. A two-sided *p* value <0.05 was considered statistically significant.

## Results

### Baseline characteristics

No statistically significant differences were observed between the two groups with respect to age, sex, ASA physical status, body mass index (BMI), or duration of surgery (*p* > 0.05) Table 1.

**Table 1:** Comparison of general information between two groups of patients [ $\bar{X}\pm S$ /case/M (Q1, Q3)].

Project	Group A (*n* = 42)	Group S (*n* = 38)	*t*/*χ*^2^/*Z* value	*p* value
Age (years)	49 ± 9	48 ± 10	0.47	0.64
Gender (male/female, example)	14/28	14/24	0.108	0.74
ASA Level I/II (example)	5/37	6/32	0.254	0.61
BMI (kg/m^2^)	23.7 ± 2.7	24.1 ± 2.5	−0.68	0.50
Surgical duration (min)	16.0 (11.0, 21.0)	15.5 (11.0, 21.3)	−0.09	0.93

Group A, alfentanil group; Group S, sufentanil group; BMI, body mass index; M (Q1, Q3), median (interquartile range).

### Incidence and severity of cough

Compared with the sufentanil group (Group S), the alfentanil group (Group A) had a significantly higher incidence of extubation-related cough (69.0% [29/42] vs. 44.7% [17/38]; unadjusted odds ratio (OR) 2.76, 95% confidence interval (CI) 1.08–7.00; *p* = 0.03). The incidence of moderate-to-severe cough during extubation was 31.0% (13/42) in Group A and 21.1% (8/38) in Group S; this difference was not statistically significant (OR 1.68; 95%CI 0.64–4.44; *p* = 0.32). Compared with the sufentanil group (Group S), the alfentanil group (Group A) had a significantly lower incidence of cough during general anesthesia induction (11.9% [5/42] vs. 31.6% [12/38]; unadjusted odds ratio (OR) 0.29, 95% confidence interval (CI) 0.10–0.89; *p* = 0.03). For the secondary endpoint of moderate-to-severe induction-related cough, the incidence was 2.4% [1/42] in the alfentanil group and 10.5% [4/38] in the sufentanil group, and this between-group difference did not reach statistical significance (OR 0.21, 95%CI 0.02–1.91; *p* = 0.36) (Tables 2, 3).

**Table 2:** Comparison of the incidence and severity of coughing during extubation under general anesthesia between two groups of patients [case (%)].

Indicator	Group A (*n* = 42)	Group S (*n* = 38)	*Z*/*χ*^2^ value	*p* value
Coughing reaction			1.82	0.07
Level 0	13 (31.0)	21 (55.3)		
Level 1	16 (38.1)	9 (23.7)		
Level 2	8 (19.0)	5 (13.2)		
Level 3	5 (11.9)	3 (7.9)		
Total cough response	29 (69.0)	17 (44.7)	4.83	0.03
Moderate to severe coughing response	13 (31.0)	8 (21.1)	1.01	0.32

Group A, alfentanil group; Group S, sufentanil group.

**Table 3:** Comparison of incidence and severity of coughing reaction during induction of general anesthesia between two groups of patients [case (%)].

Indicator	Group A (*n* = 42)	Group S (*n* = 38)	*Z*/*χ*^2^ value	*p* value
Coughing reaction			2.22	0.03
Level 0	37 (88.1)	26 (68.4)		
Level 1	4 (9.5)	8 (21.1)		
Level 2	1 (2.4)	3 (7.9)		
Level 3	0 (0)	1 (2.6)		
Total cough response	5 (11.9)	12 (31.6)	4.62	0.03
Moderate to severe coughing response	1 (2.4)	4 (10.5)	_^a^	0.36

Group A, alfentanil group; Group S, sufentanil group.

^a^Fisher’s exact test.

### Hemodynamic parameters

**Figure 2:**
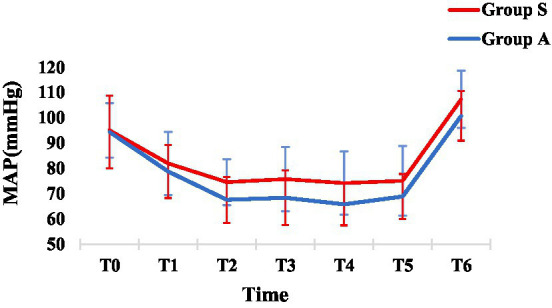
Comparison of mean arterial pressure (MAP) between the two groups at T0–T6; specify that error bars represent standard deviation (SD); Group A, alfentanil group; Group S, sufentanil group; T0, upon arrival in the operating room; T1, before intubation; T2, 1 min after intubation; T3, 3 min after intubation; T4, 1 min after suspension laryngoscope placement; T5, 3 min after suspension laryngoscope placement; T6, at extubation.

**Figure 3:**
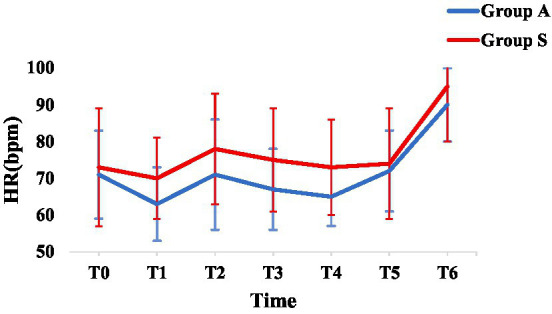
Comparison of heart rate (HR) between the two groups at T0–T6; specify that error bars represent standard deviation (SD); Group A, alfentanil group; Group S, sufentanil group; T0, upon arrival in the operating room; T1, before intubation; T2, 1 min after intubation; T3, 3 min after intubation; T4, 1 min after suspension laryngoscope placement; T5, 3 min after suspension laryngoscope placement; T6, at extubation.

Box’s M test indicated that the covariance matrices were not homogeneous for repeated measures analysis of variance (ANOVA) (mean arterial pressure [MAP]: *F* = 6.99, *p* < 0.001; heart rate [HR]: *F* = 2.15, *p* < 0.001). Therefore, we used the multivariate test (Pillai’s trace), as this method is robust to heterogeneity of covariance matrices. The results of sensitivity analysis using linear mixed-effects models were consistent with those of the multivariate test, confirming the robustness of our findings. Multivariate tests demonstrated a statistically significant effect of time on both MAP and HR (both *p* < 0.001), indicating significant within-subject variation across time points when the two groups were analyzed as a whole. A significant between-group effect was observed for both MAP (*F* = 7.04, *p* = 0.01) and HR (*F* = 7.44, *p* = 0.01). The time-by-group interaction was not statistically significant for MAP (*F* = 0.71, *p* = 0.43) but was statistically significant for HR (*F* = 2.52, *p* = 0.03). Simple effects analysis was performed subsequently. For pairwise between-group comparisons at fixed time points, we applied Bonferroni correction for multiple comparisons. Accordingly, all *p* values reported below are Bonferroni-adjusted *p*-values. For MAP, between-group comparisons at fixed time points revealed that MAP was significantly lower in Group A than in Group S from T2 to T5 (all *p* < 0.05). Within-group analysis showed that MAP in both groups remained at lower levels from T2 to T5. For HR, between-group comparisons showed that HR was significantly lower in Group A than in Group S from T1 to T4 (all *p* < 0.05). Within-group analysis indicated that HR in both groups decreased at T1, increased and stabilized from T2 to T5, and then increased significantly above baseline at T6 (Table 4) (Figures 2, 3).

**Table 4:** Comparison of MAP and HR between two groups of patients at different time points ( $\bar{X}\pm S$ ).

Times	MAP (mmHg)		HR (bpm)	
	Group A (*n* = 42)	Group S (*n* = 38)	Group A (*n* = 42)	Group S (*n* = 38)
T0	94 ± 14	95 ± 11	71 ± 12	73 ± 16
T1	79 ± 10	82 ± 12	63 ± 10^*^	70 ± 11
T2	68 ± 9^*^	75 ± 9	71 ± 15^*^	78 ± 15
T3	68 ± 11^*^	76 ± 13	67 ± 11^*^	75 ± 14
T4	66 ± 8^*^	74 ± 12	65 ± 8^*^	73 ± 13
T5	69 ± 9^*^	75 ± 14	72 ± 11	74 ± 15
T6	101 ± 10	107 ± 11	90 ± 10	95 ± 15

Values are presented as mean ± standard deviation.

Group A, Alfentanil group; Group S, Sufentanil group; T0, upon arrival in the operating room; T1, before intubation; T2, 1 min after intubation; T3, 3 min after intubation; T4, 1 min after suspension laryngoscope placement; T5, 3 min after suspension laryngoscope placement; T6, at extubation; MAP, mean arterial pressure; HR, heart rate. Statistical results from repeated measures ANOVA for MAP: Between-group effect, *F* = 7.04, *p* = 0.01; Time effect, *F* = 11.701, *p* < 0.001; Group × Time interaction, *F* = 0.71, *p* = 0.43. Statistical results from repeated measures ANOVA for HR: Between-group effect, *F* = 7.44, *p* = 0.01; Time effect, *F* = 7.418, *p* < 0.001; Group × Time interaction, *F* = 2.52, *p* = 0.03.

^*^p < 0.05 versus Group S at the same time point, adjusted by Bonferroni correction for multiple comparisons.

### Postoperative recovery

No statistically significant differences were observed between the two groups in extubation time, modified Aldrete scores on admission to or discharge from the post-anesthesia care unit (PACU), or Numerical Rating Scale (NRS) pain scores at 1 h postoperatively (*p* > 0.05) (Table 5).

**Table 5:** Postoperative recovery outcomes in the two groups [M (Q1, Q3)].

Indicator	Group A (*n* = 42)	Group S (*n* = 38)	*Z*/*χ*^2^/*t* value	*p* value
Extubation time (min)	11 (9.5, 15)	12 (10, 14)	−0.50	0.62
Aldrete score on PACU admission (points)	8 (8, 9)	8 (8, 9)	0.00	0.99
Aldrete score out PACU admission (points)	10 (10, 10)	10 (10, 10)	0.000	1.000
Postoperative 1-h NRS score (points)	1 (0, 1.5)	1 (0, 2)	−0.30	0.76

Group A, alfentanil group; Group S, sufentanil group; NRS, numerical rating scale.

### Adverse events

In terms of safety outcomes, no treatment-related serious adverse events (SAEs) were reported in either the alfentanil (Group A) or sufentanil (Group S) group; predefined SAEs of interest included postoperative surgical site bleeding, laryngospasm/bronchospasm, severe respiratory depression, opioid-induced chest wall rigidity, intractable pruritus, and acute urinary retention. The overall incidence of postoperative nausea and vomiting (PONV) was 9.5% [4/42] in Group A and 13.2% [5/38] in Group S, There was no statistically significant difference in the incidence of PONV between the two groups (Fisher’s exact test, two-sided *p* = 0.742).

## Discussion

In this randomized controlled trial involving patients undergoing suspension laryngoscopy surgery, the results demonstrate that alfentanil, compared with sufentanil, significantly increases the incidence of cough during tracheal extubation (69.0% vs. 44.7%; OR = 2.76; 95%CI, 1.08–7.00; *p* = 0.03) but does not increase the risk of moderate-to-severe cough (31.0% vs. 21.1%; *p* = 0.32). Conversely, during induction of general anesthesia, alfentanil exhibits a clear advantage with a lower incidence of cough (11.9% vs. 31.6%; OR = 0.29; 95%CI, 0.10–0.89; *p* = 0.03). In addition, our study findings demonstrated that alfentanil achieved more effective mitigation of the hemodynamic stress responses triggered by tracheal intubation and suspension laryngoscope placement, compared with sufentanil. Meanwhile, no statistically significant between-group differences were observed in key secondary recovery and safety outcomes, including extubation time, postoperative numerical rating scale (NRS) pain scores, and overall incidence of adverse events.

While suspension laryngoscopy is classified as a minimally invasive otolaryngologic procedure, intraoperative airway instrumentation and rigid laryngoscope placement deliver intense noxious stimulation to the pharyngeal and tracheal mucosa, creating a high-risk setting for perioperative cough reflex activation. Cough during anesthesia emergence and tracheal extubation is one of the most prevalent adverse events in this surgical cohort, with existing literature reporting an incidence ranging from 50% to 70% (1). Beyond airway irritation, sustained and severe coughing drives acute pathological elevations in intracranial pressure, intraocular pressure, and systemic arterial pressure, directly increasing the risk of life-altering complications including surgical site bleeding, refractory laryngospasm, and acute cardiovascular events (3).

Sufentanil, a high-affinity selective *μ*-opioid receptor agonist, is widely used for perioperative analgesia due to its dose-dependent efficacy and favorable hemodynamic stability. Nevertheless, sufentanil-induced cough remains a well-documented clinical concern, with prior studies reporting an incidence as high as 31.9% during induction (9). Previous investigations have largely focused on the suppressive effect of alfentanil on opioid-induced cough in the induction setting (10, 11)^.^ This body of evidence is consistent with the principal finding of the present study: a significantly lower incidence of induction-related cough in the alfentanil cohort compared with the sufentanil cohort. The protective effect is likely attributable to the rapid pharmacokinetic onset of alfentanil, which enables swift occupancy of opioid receptors in the medullary cough center and at peripheral sites—including the superior laryngeal nerve and pulmonary vascular endothelium—thereby interrupting both the afferent and efferent limbs of the cough reflex arc (12).

The differential incidence of cough observed across anesthetic phases may be attributed to the pharmacokinetic profile of alfentanil. Alfentanil exhibits low lipid solubility and a very short plasma-effect site equilibration half-life (0.6–1.0 min), enabling it to rapidly achieve a high effect-site concentration during induction (13). Lee et al. (14) demonstrated that an alfentanil dose of ≥14 μg·kg^−1^ effectively suppresses cough during extubation. In contrast, during the emergence and extubation phases, the plasma concentration of alfentanil declines exponentially after infusion cessation. The decrease in its therapeutic threshold concentration occurs more rapidly than that of sufentanil (15). Schraag et al. (16) found that the fractional decrease in plasma concentration required for extubation was significantly lower for alfentanil (48.0%) than for sufentanil (62.1%), suggesting that the threshold for airway reflex recovery is more readily reached with alfentanil. At the mechanistic level, Ahonen et al. (17) directly confirmed that the time for an 80% decrease in plasma concentration (t_80_) after stopping the infusion was significantly shorter for alfentanil than for sufentanil, and that t80 was linearly correlated with extubation time. Collectively, these lines of evidence support our hypothesis that the rapid decline in alfentanil’s effect-site concentration allows earlier recovery of airway protective reflexes, which, when confronted with extubation stimuli, manifests as a higher incidence of cough—the so-called “rebound” phenomenon. This finding complements the work of Li et al. (18), who reported only a reduction in cough during induction in ambulatory surgery without evaluating the extubation phase, thereby highlighting the heterogeneous cough response associated with alfentanil across different stages of general anesthesia. Current clinical strategies for mitigating cough responses include topical lidocaine application (3), intravenous lidocaine or dexmedetomidine (19), and laryngeal nerve blocks (20).

In this study, alfentanil was associated with lower MAP and HR values at several intraoperative time points. For MAP, the time-by-group interaction did not reach statistical significance, indicating that the overall pattern of MAP change over time was similar between the two groups; the observed differences at individual time points may reflect exploratory findings rather than a consistent superior effect. For HR, in contrast, a significant time-by-group interaction was observed, providing stronger support for a differential hemodynamic profile between the two groups. Collectively, these results suggest that alfentanil may offer some advantages in blunting the hemodynamic responses to intubation and laryngoscope placement, but this conclusion is more robust for HR than for MAP. This finding aligns with the study by Farzi et al. (21) Since this study did not monitor the plasma concentrations of the alfentanil and sufentanil, we speculate that this may be attributed to alfentanil’s rapid onset and prompt achievement of peak plasma concentration, which facilitates swift attenuation of the intubation stress response. No significant differences were observed between the two groups in extubation time, Aldrete scores on PACU admission and discharge, or NRS pain scores at 1 h postoperatively (*p* > 0.05). This negative result is expected and suggests that both opioids provide satisfactory recovery quality in the context of short-duration ambulatory surgery. Suspension laryngoscopy is associated with minimal surgical trauma and mild postoperative pain; thus, differences in analgesic potency between the two opioids were not reflected in the NRS scores. Although the incidence of cough during extubation was higher in the alfentanil group, this did not translate into prolonged extubation time or extended PACU stay. This likely reflects that most coughing episodes were mild (Grade 1) and transient, resolving promptly after extubation without clinically meaningful impact on overall emergence quality.

In this limited, low-risk study population, no serious adverse events related to opioids, including respiratory depression and chest wall rigidity, were observed. Despite the higher frequency of coughing during extubation in the alfentanil group, this did not result in surgical wound bleeding or severe airway spasm. As the study excluded patients with ASA physical status ≥ III or severe cardiopulmonary dysfunction, and based on the findings reported by Chen et al. (11), the safety of the dosage regimen employed in this study (induction 21 μg/kg; maintenance 0.7–1.4 μg·kg^−1^·min^−1^) in higher-risk patients remains to be determined. The choice of alfentanil and sufentanil doses was based on an estimated potency ratio of 1:70 derived from classical pharmacology literature. However, we acknowledge that precise equianalgesic dosing between different opioids for the specific endpoint of cough suppression remains undefined. This inherent limitation in opioid comparator studies should be considered when interpreting our results.

Limitations: This study has several limitations. First, the sample size calculation was based on the incidence of any cough (Grades 1–3), not moderate-to-severe cough (Grades 2–3). Therefore, the negative finding for moderate-to-severe cough should be interpreted with caution, as the study may not have been adequately powered to detect smaller yet clinically relevant differences in cough severity. This limitation applies to both the primary and secondary cough severity endpoints. Second, the study evaluated only a single fixed-dose regimen; therefore, the optimal dose of alfentanil for suspension laryngoscopy surgery remains undetermined. Third, effect-site concentrations of the opioids were not monitored; drug administration was guided solely by clinical signs and bispectral index (BIS) values, which may have influenced the results. Fourth, the severity of coughing was assessed using a semi-quantitative grading scale, which, despite being evaluated by blinded investigators, cannot completely avoid subjectivity. Fifth, long-term follow-up was not conducted; consequently, the impact of the two opioids on long-term outcomes, such as chronic cough, remains unknown.

## Conclusion

In patients undergoing suspension laryngoscopy surgery, alfentanil is associated with a lower incidence of cough during induction of general anesthesia but a higher incidence of cough during tracheal extubation compared with sufentanil. There was no statistically significant difference in the incidence of moderate-to-severe cough between alfentanil and sufentanil. If the clinical priority is to minimize the risk of cough during emergence and extubation, sufentanil may remain the more prudent choice.

## Data Availability

The datasets presented in this article are not readily available because data are not publicly available because they contain information that could compromise the privacy of study participants. Access may be granted only to qualified researchers upon reasonable request and with appropriate ethical approvals. Requests to access the datasets should be directed to Wei Qqiang, wq19831363019@163.com.

## Footnotes

1
https://www.chictr.org.cn/

## References

[1] Arslan IB Kose I Ciger E Demirhan E Gumussoy M Cukurova I. Does topical anesthesia using aerosolized lidocaine inhibit the superior laryngeal nerve reflex? Otolaryngol Head Neck Surg. (2013) 149:466–72. doi: 10.1177/0194599813495372, 23818488

[2] Akhondzadeh R Olapour A Rashidi M Elyasinia F. Comparison of sedation with dexmedetomidine-alfentanil versus ketamine-alfentanil in patients undergoing closed reduction of nasal fractures. Anesth Pain Med. (2020) 10:e102946. doi: 10.5812/aapm.102946, 33134144 PMC7539046

[3] Lv L Yan L Liu X Chen M. Effectiveness of lidocaine/prilocaine cream on cardiovascular reactions from endotracheal intubation and cough events during recovery period of older patients under general anesthesia: prospective, randomized placebo-controlled study. BMC Geriatr. (2020) 20:157. doi: 10.1186/s12877-020-01567-y, 32366224 PMC7197116

[4] Ghodraty MR Hasani V Bagheri-Aghdam A Zamani MM Pournajafian A Rokhtabnak F . Remifentanil infusion during emergence moderates hemodynamic and cough responses to the tracheal tube. J Clin Anesth. (2016) 33:514–20. doi: 10.1016/j.jclinane.2015.09.001 26603110

[5] White LD Hodsdon A An GH Thang C Melhuish TM Vlok R. Induction opioids for caesarean section under general anaesthesia: a systematic review and meta-analysis of randomised controlled trials. Int J Obstet Anesth. (2019) 40:4–13. doi: 10.1016/j.ijoa.2019.04.007, 31230994

[6] Wang J Xu X Wang Z Xu Q Zou X Wu J . Low-dose alfentanil inhibits sufentanil-induced cough during anesthesia induction: a prospective, randomized, double-blind study. Drug Des Devel Ther. (2023) 18:1603–12. doi: 10.2147/DDDT.S464823, 38774482 PMC11108069

[7] Evers AS Crowder CM Balser JR Minogue SC Ralph J Lampa MJ . "General anesthetics". In: Brunton LL Lazo JS Parker KL, editors. Goodman and Gilman's the Pharmacological Basis of Therapeutics, 11th Edn. New York: McGraw-Hill (2006). p. 341–68.

[8] Minogue SC Ralph J Lampa MJ. Laryngotracheal topicalization with lidocaine before intubation decreases the incidence of coughing on emergence from general anesthesia. Anesth Analg. (2004) 99:1253–7. doi: 10.1213/01.ANE.0000132779.27085.52, 15385385

[9] Liu XS Xu GH Shen QY Zhao Q Cheng XQ Zhang J . Dezocine prevents sufentanil-induced cough during general anesthesia induction: a randomized controlled trial. Pharmacol Rep. (2015) 67:52–5. doi: 10.1016/j.pharep.2014.08.004, 25560575

[10] Wang L Wu Q Wang M Ming W Sheng C Zhang Y . The safety and efficacy of alfentanil combined with midazolam in fiberoptic bronchoscopy sedation: a randomized, double-blind, controlled trial. Front Pharmacol. (2022) 13:1036840. doi: 10.3389/fphar.2022.1036840, 36339547 PMC9634630

[11] Chen X Han M Shu A Zhou M Wang K Cheng C. Effects of different doses of alfentanil on cardiovascular response to rapid sequence intubation in elderly patients: a parallel-controlled randomized trial. BMC Anesthesiol. (2024) 24:290. doi: 10.1186/s12871-024-02663-x, 39138407 PMC11320851

[12] Lee MG Chang YJ Park JM Park HY. The clinical effective dose of alfentanil for suppressing cough during emergence from desflurane anesthesia. Korean J Anesthesiol. (2011) 61:292–6. doi: 10.4097/kjae.2011.61.4.292, 22110881 PMC3219774

[13] Brosnan RJ Pypendop BH Stanley SD. Phenylpiperidine opioid effects on isoflurane minimum alveolar concentration in cats. J Vet Pharmacol Ther. (2020) 43:533–7. doi: 10.1111/jvp.12886, 32557697

[14] Lee MG Chang YJ Park JM Park HY. The clinical effective dose of alfentanil for suppressing cough during emergence from desflurane anesthesia. Korean J Anesthesiol. (2011) 61:292–6. doi: 10.4097/kjae.2011.61.4.292, 22110881 PMC3219774

[15] Park S Kim JH Bae JC Lee JR Kim MS. Tracheal intubation with or without a neuromuscular blocking agent for a short surgical procedure in children: prospective, randomized, double-blind trial. Paediatr Anaesth. (2021) 31:863–70. doi: 10.1111/pan.14205, 33993571

[16] Schraag S Mohl U Hirsch M Stolberg E Georgieff M. Recovery from opioid anesthesia: the clinical implication of context-sensitive half-times. Anesth Analg. (1998) 86:184–90. doi: 10.1097/00000539-199801000-00036, 9428876

[17] Ahonen J Olkkola KT Hynynen M Seppälä T Ikävalko H Remmerie B . Comparison of alfentanil, fentanyl and sufentanil for total intravenous anaesthesia with propofol in patients undergoing coronary artery bypass surgery. Br J Anaesth. (2000) 85:533–40. doi: 10.1093/bja/85.4.533, 11064610

[18] Li H Zhang H Cheng Z Ji H. Effects of alfentanil hydrochloride on cough and hemodynamics during induction of general anesthesia in daytime surgery. Minerva Surg. (2023) 78:730–2. doi: 10.23736/S2724-5691.21.09341-2, 35088992

[19] Hu S Li Y Wang S Xu S Ju X Ma L. Effects of intravenous infusion of lidocaine and dexmedetomidine on inhibiting cough during the tracheal extubation period after thyroid surgery. BMC Anesthesiol. (2019) 19:66. doi: 10.1186/s12871-019-0739-1, 31054568 PMC6500031

[20] Binhazzaa A. Efficacy and safety of superior laryngeal nerve block in neurogenic cough: a systematic review and meta-analysis. Eur Arch Otorrinolaringol. (2025) 282:5209–18. doi: 10.1007/s00405-025-09582-8, 40900321

[21] Farzi F Mehrafza M Mirmansouri A Sorouri ZZ Roushan ZA Raoufi A . Hemodynamic parameters and reproductive outcome after intracytoplasmic sperm injection and fresh embryo transfer in patients undergoing oocyte retrieval with general anesthesia using fentanyl, remifentanil or alfentanil—a randomized clinical trial. Taiwan J Obstet Gynecol. (2019) 58:536–40. doi: 10.1016/j.tjog.2019.05.019, 31307747

